# A Fiberglass-Cloth-Reinforced Perfluorosulfonic Acid Membrane

**DOI:** 10.3390/membranes15060166

**Published:** 2025-06-02

**Authors:** Zhutao Zhang, Yiru Dou, Wen Zhang, Li Xu, Yuxin Wang

**Affiliations:** 1 State Key Laboratory of Chemical Engineering and Low-Carbon Technology, Tianjin Key Laboratory of Membrane Science and Desalination Technology, School of Chemical Engineering and Technology, Tianjin University, Tianjin 300072, China; zhangzhutao@tju.edu.cn (Z.Z.); douyiru@tju.edu.cn (Y.D.); xuli620@tju.edu.cn (L.X.); 2 National Industry-Education Integration Platform of Energy Storage, Tianjin University, Tianjin 300072, China

**Keywords:** perfluorosulfonic acid, solution casting, fiberglass cloth, composite membrane

## Abstract

Perfluorosulfonic acid (PFSA) membranes have found broad-ranging applications, owing to their high ionic conductivity and excellent chemical stability. However, membranes with higher mechanical strength, lower area-specific resistance, reduced swelling, less gas crossover and more affordable costs are desirable. Herein, we report on the fabrication of a fiberglass-cloth-reinforced PFSA membrane using a simple solution cast method. The breaking strength of the reinforced membrane has the potential to reach 81 MPa, which is about 6 times and 2.5 times that of its non-reinforced counterpart and the commercial Nafion 117 (N117) membrane, respectively. The area swelling ratio of the reinforced membrane is lowered to merely 3%, which is only about 1/12 that of N117, in water at 100 °C. Despite ionic conduction being hindered by the fiberglass cloth, the reinforced PFSA membrane shows an area-specific resistance of only 0.069 Ω·cm^2^, which is 58% lower than that of N117, under 80 °C and 100% humidity. This research provides a promising technological pathway for the development of high-performance ionic conductive membranes.

## 1. Introduction

Perfluorosulfonic acid (PFSA) is a cation-conducting resin composed of polytetrafluoroethylene (PTFE) backbones and perfluorovinyl ether side chains with sulfonate ionic end groups [1,2,3]. Since its initial launch by DuPont under the trademark name Nafion in the late 1960s, PFSA, commonly used in the form of a membrane, has attracted extensive academic interest and achieved a wide range of industrial applications. This is primarily attributed to its unparalleled high ion conductivity, chemical resistance, and thermal stability [1,2,4,5], which have seldom been matched by subsequent materials so far. Following its substantial contribution to the advancement of the chlor-alkali industry, PFSA currently plays a key role in the fields of energy storage and conversion, which are critical for addressing the urgent issue of global warming. This is exemplified by the utilization of PFSA electrolyte membranes in fuel cells [6,7], water electrolysis [8,9,10], and flow batteries [11,12,13].

Although it possesses a range of desirable properties for electrochemical devices, PFSA membranes are not without their limitations. PFSA membranes exhibit low area-specific resistance (ASR) and high ion conductivity but only under conditions of sufficient hydration [14,15]; the latter, however, can cause significant dimensional changes to the membrane, e.g., high swelling [16]. Excessive swelling of an electrolyte membrane can result in several adverse effects. One such effect is the internal generation of uneven and alternating stress, which can accelerate crack formation and thus reduce the mechanical stability of the membrane [17,18,19]. Additionally, a high swelling ratio in the membrane facilitates rapid molecular crossover, leading to a lower purity of gases being produced in water electrolyzers [20] or reduced fuel efficiency in direct methanol fuel cells [21]. Furthermore, the catalyst layer in a membrane electrode assembly (MEA) may partially delaminate more easily when the membrane exhibits high swelling, resulting in the accelerated performance degradation of electrolytic cells [22,23].

In order to prevent excessive swelling and enhance the dimensional stability of PFSA membranes, numerous strategies have been proposed. Various methods of chemical cross-linking, often at a cost to ionic conducting sites, have been attempted [20,24,25,26]; however, to date, they have not achieved much success. Different hydrophobic polymers have also been used for blending with PFSA to suppress membrane swelling [27], but this has often led to either unsatisfactory swelling suppression or unacceptable restrictions to ionic conduction. Furthermore, researchers have attempted to reinforce PFSA membranes by dispersing various particles [24,28,29], platelets [30,31] or fibers [32,33] inside PFSA matrix. In contrast, “pore-filling” methods have been used to enhance the mechanical strength and dimensional stability of PFSA membranes by filling PFSA resin into the pores of various microporous matrices, including nonwoven fiber networks [34,35], porous polymer membranes [19,36,37] and porous ceramic membranes [38,39]. Among the different pore-filling membranes, a membrane trademarked as Gore-Select has attained remarkable commercial success and become the dominate proton exchange membrane (PEM) in PEM fuel cells. The Gore-Select membrane is fabricated by impregnating a microporous membrane of expanded PTFE (ePTFE) with PFSA [40]. The membrane shows a swelling ratio that is lowered to a great extent, owing to the restriction imposed by the high-strength ePTFE membrane. It also maintains a low ASR, which is enabled by the greatly reduced membrane thickness to compensate for decreased conductivity.

Herein, we report on the fabrication of a PFSA membrane reinforced with fiberglass cloth (FGC). FGC, a woven fabric, is known for its excellent mechanical strength, corrosion resistance, high-temperature resistance and insulation properties. It is usually used as a strengthening material for polymer resins and has found applications across various domains. A variety of FGCs are commercially available, these being the most cost-effective among all high-strength fabrics. In this research, FGC-reinforced PFSA membranes were fabricated using a straightforward solution casting method. The membrane’s ion conductivity, ASR, mechanical strength, and swelling properties were tested. Comparisons were also made with unreinforced-solution-casted PFSA membranes and commercial Nafion membranes.

## 2. Experimental

### 2.1. Materials and Chemicals

Perfluorosulfonic acid resin powder (PFSA, 1.03 mmol/g, 1.98 g/cm^3^) was purchased from Shandong Dongyue Future Hydrogen Energy Material Co., Ltd. (Zibo, China). Fiberglass cloth (FGC, 17 g/m^2^, 19 g/m^2^) was obtained from Hangzhou Textile Composite Material Co., Ltd. (Hangzhou, China). Nafion membranes (N212, N117) were purchased from Chemours Chemistry (Shanghai) Co., Ltd. (Shanghai, China). N, N-dimethylformamide (DMF, AR) and ferrous sulfate heptahydrate FeSO_4_·7H_2_O (AR) were purchased from Tianjin Kermel Chemical Reagents Co., Ltd. (Tianjin, China). Trichloromethane CHCl_3_ (AR) and absolute ethyl alcohol C_2_H_5_OH (AR) was purchased from Tianjin Yuanli Chemical Co., Ltd. (Tianjin, China). Hydrogen peroxide H_2_O_2_ (AR, 30 wt.%) was obtained from Tianjin Jiangtian Chemical Technology Co., Ltd. (Tianjin, China). Sulfonic acid H_2_SO_4_ (AR, 98%) was obtained from Rionlon (Tianjin) Pharmaceutical Chemistry Co., Ltd. (Tianjin, China). Hydrogen H_2_ (99.999%) and nitrogen N_2_ (99.999%) gases were purchased from Tianjin Boliming Technology Co., Ltd. (Tianjin, China). Deionized water (>1 MΩ·cm) was obtained from Tianjin Yongqingyuan Distilled Water Shop (Tianjin, China).

### 2.2. Membrane Fabrication

Two kinds of FGC, G17 and G19, with gram weights of 17 g/m^2^ and 19 g/m^2^, respectively, were used in this study. The received FGCs were washed successively with trichloromethane, ethyl alcohol and deionized water before drying for future use. A 5 wt.% PFSA DMF solution was prepared by dissolving 1 g PFSA powder into 19 g DMF under reflux at 120 °C for 2 h. The solution was cooled to room temperature and drop-coated on an FGC, which was spread flat on a glass plate. The coated FGC was allowed to stand for 12 h to ensure full solution impregnation and then dried at 80 °C for 6 h. The dried PFSA was immersed in water and peeled off from the glass plate to obtain the FGC reinforced PFSA membrane. The PFSA membranes reinforced with G17 and G19 are signified as PFSA/G17 and PFSA/G19, respectively. For comparison purposes, a pure-PFSA membrane without FGC was also cast under identical conditions, and it is signified as P-PFSA.

### 2.3. Characterizations and Property Tests

The morphology of the membrane was imaged using a scanning electron microscope (SEM, 8100, Hitachi, (Tokyo, Japan)), with the membrane’s cross-section exposed via cryo-fracture in liquid nitrogen. Infrared spectra of the membrane were recorded using an attenuated total reflection Fourier-transform infrared (ATR-FTIR) spectrophotometer (FTIR-650, Tianjin Gangdong Sci. &Tech. Co., Ltd., (Tianjin, China)). Thermogravimetric analysis (TGA) was conducted using a thermal analyzer (STA300, Hitachi) at a heating rate of 10 °C/min. under a N_2_ atmosphere. The hydrophilicity of the membrane was tested using a contact angle meter (OCA15EC, Dataphysics, (Filderstadt, Germany)).

The proton conductivity and area specific resistance of the membrane were measured using electrochemical impedance spectroscopy (EIS). Prior to the measurement, the membranes to be tested were treated by immersing them successively in 5 wt.% H_2_O_2_ solution, 1M H_2_SO_4_ solution and deionized water at 80 °C for 1 h. The treated membranes were fixed inside a membrane test system (MTS-740, Scribner, (Southern Pines, NC, USA)) with a four-electrode configuration. The test system was connected to an electrochemical workstation (Parstat 2273, Princeton Applied Research, (Oak Ridge, TN, USA)), which exerted an oscillating voltage of 10 mV at a frequency from 100 Hz to 1 MHz and outputted an impedance spectrum in the form of a Nyquist plot. The software Z-view 3.1 was used in the fitting of the spectrum to obtain the membrane resistance, R, from which the area-specific resistance, $R_{A}$, and conductivity, σ, were calculated according to the following:

(1) $${\;R}_{A}=R\times A,\;\sigma =l/(R\times A)$$

where l and A are the thickness and effective area (0.5 cm^2^) of the membrane.

The tensile strength of the membrane at ambient temperature was measured using a mechanical testing machine (ZCW-5000, Jinan Zhongchuang Industrial Test System, (Jinan, China)), with an elongation rate of 5 mm min^−1^. The tensile specimen was cut from the membrane into a dumbbell shape, with its central portion being a 4 mm × 50 mm strip.

The water uptake and swelling ratios of the membrane were calculated according to the changes in the membrane in mass, area and thickness between its dry and fully hydrated states:

(2) $$WU\left(\%\right)=\frac{W_{wet}-W_{dry}}{W_{dry}}\times 100$$

(3) $$A_{SR}\left(\%\right)=\frac{A_{wet}-A_{dry}}{A_{dry}}\times 100$$

(4) $$T_{SR}\left(\%\right)=\frac{T_{wet}-T_{dry}}{T_{dry}}\times 100$$

where ${\;W}_{dry}$, $A_{dry}$ and $T_{dry}$ are, respectively, the mass, area and thickness of the membrane after being dehydrated at 80 °C for 24 h, while $W_{wet}$, $A_{wet}\;$ and $T_{wet\;}$ are, respectively, the membrane’s mass, area and thickness when it reaches equilibrium in deionized water at a given temperature. Three membrane samples were used in each condition, and the results were averaged.

The hydrogen gas permeation flux $J_{H}$ and permeability $K_{H}$ of the membrane were measured using an electrochemical method described in Ref. [41]. The membrane to be tested was sandwiched between two identical Pt/C electrodes, and H_2_ and N_2_ gases were flowing through the cathode and anode, respectively. The H_2_ gas that permeated through the membrane from the cathode side was oxidized at the anode. Under a high-enough positive potential, the oxidation current will be limited by the speed of H_2_ permeation, so this limiting current was used to calculate $J_{H}$ and $K_{H}$ via the following equation:

(5) $$J_{H}=\frac{J_{L}}{2F}$$

(6) $$K_{H}=\frac{J_{L}\times \delta_{m}}{2F\times P_{H}}$$

where ${\;J}_{L}$ is the limiting current density, $\delta_{m}$ is the membrane thickness, $P_{H}$ is the pressure of the cathode side, and F is the Faraday constant.

The membranes or FGC samples were immersed in Fenton’s reagent containing 3 wt.% H_2_O_2_ and 40 ppm Fe^2+^ (which come from FeSO_4_·7H_2_O) at ambient temperature. After immersing them for different times, the samples were removed from the reagent, washed with deionized water, dried at 80 °C for 4h and weighed. The weight obtained, together with the initial weight, was used to calculate the samples’ remaining weight in a percentage at a given immersing time.

## 3. Results and Discussions

**Figure 1:**
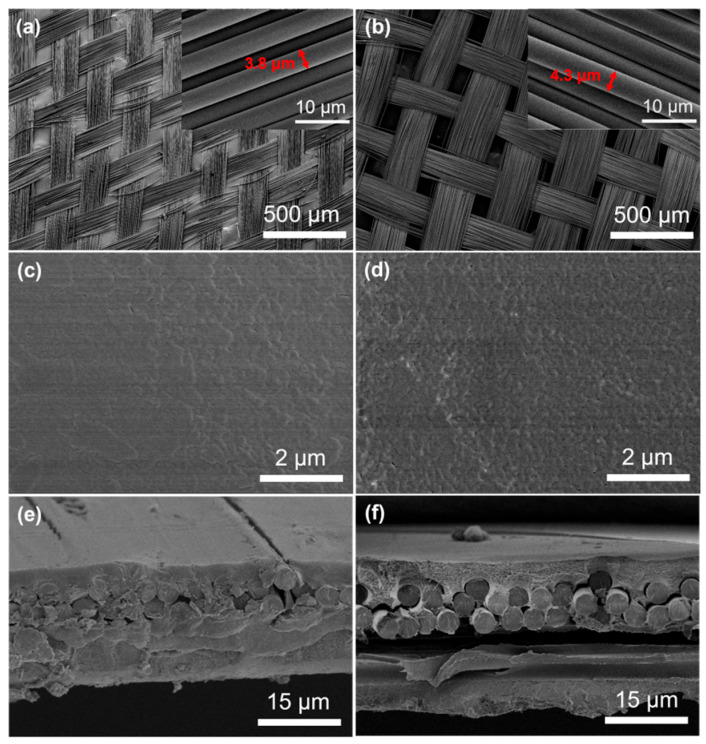
SEM images: (**a**) the top view of the G17 fiberglass cloth, (**b**) the top view of the G19 fiberglass cloth, (**c**) the surface view and (**e**) cross-sectional view of the PFSA/G17 membrane, and (**d**) the surface view and (**f**) cross-sectional view of the PFSA/G19 membrane.

The morphologies of G17 and G19 fiberglass cloth and the membranes PFSA/G17 and PFSA/G19 are shown in Figure 1. It is shown that the FGC used was loosely woven by yarns consisting single glass fibers measuring <5 μm in diameter (Figure 1a,b, insert). This mesh-like fabric is desirable in reinforcing the PFSA membrane, since the mesh holes should facilitate ion conduction. The straightforward fabrication by solution casting resulted in a composite membrane, PFSA/FGC, with a dense and flat surface (Figure 1c,d). Moreover, the FGC appeared to be firmly bonded with the surrounding PFSA (Figure 1e), probably because of the affinity between the polar groups in the two specimens.

**Figure 2:**
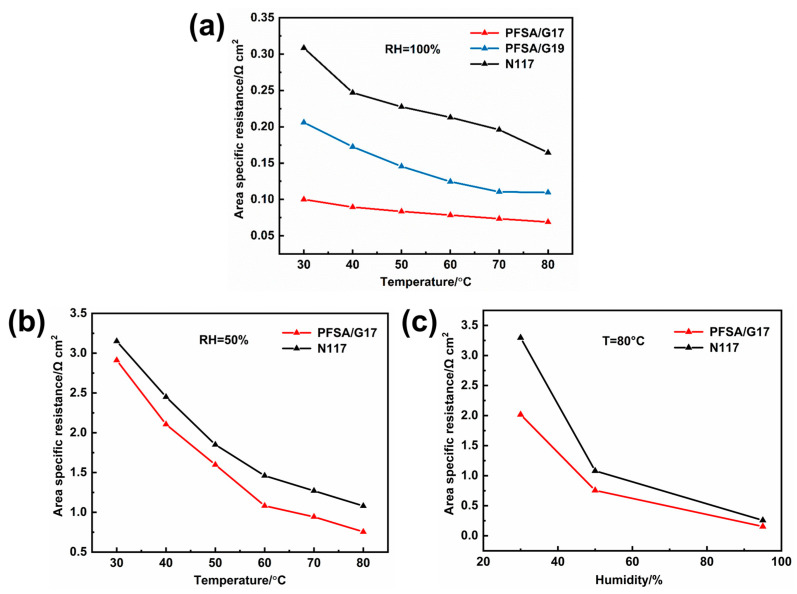
(**a**) ASR vs. temperature at 100% relative humidity for PFSA/G17, PFSA/G19 and N117 membranes; (**b**) ASR vs. temperature at 50% relative humidity for PFSA/G17 and N117 membranes; (**c**) ASR vs. humidity at 80 °C for PFSA/G17 and N117 membranes.

Although the incorporation of FGC led to a reduction in ion conductivity (Figure S1a), the FGC-reinforced PFSA membranes showed a significantly lower ASR compared to the Nafion 117 membrane (N117) (Figure 2a and Figure S1b). Notably, PFSA/G17 exhibited an ASR of merely 0.069 Ω·cm^2^, which is 58% lower than that of N117, under the normal operating conditions of 80 °C and 100% relative humidity for proton exchange membrane fuel cells. The substantial differences in ASR can be attributed mainly to the changes in membrane thickness in their hydrated state from 210 μm of N117 to 25 μm of PFSA/G19 and 25 μm of PFSA/G17. PFSA/G17 also outperformed N117 in terms of ASR under low-humidity conditions (Figure 2b,c). Furthermore, PFSA/G17 exhibited higher ion conductivity retention, as its ASR did not increase as sharply with decreasing humidity (Figure 2c). Presumably, the higher conductivity retention of PFSA/G17 is related to the water affinity of FGC, which can alleviate water loss when humidity decreases and thus prevent a sharp increase in ASR.

**Figure 3:**
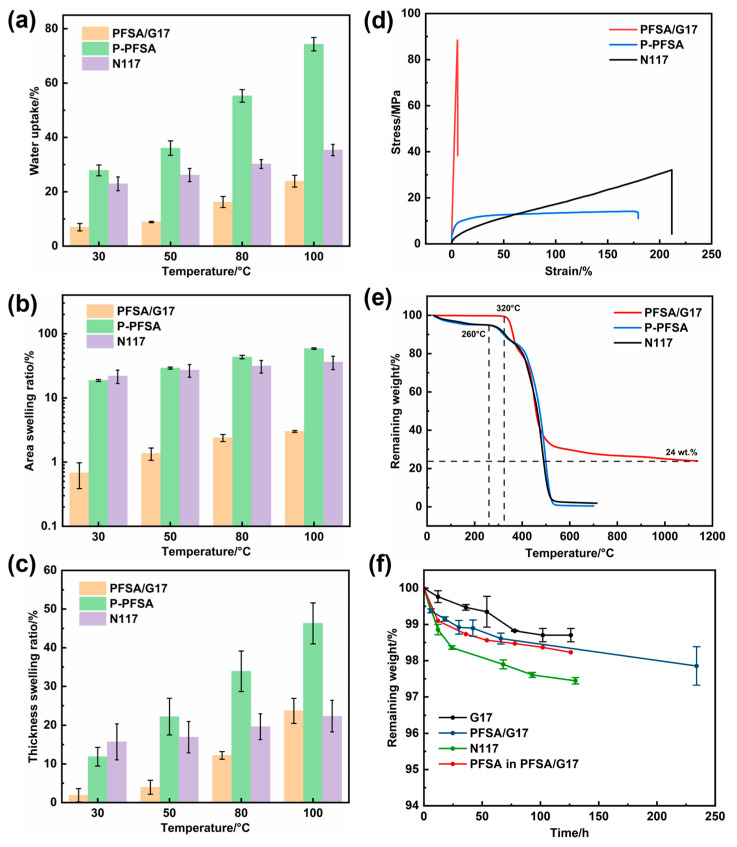
The swelling properties of the three membranes: (**a**) water uptakes, (**b**) area swelling ratios, and (**c**) thickness swelling ratios; (**d**) Stress–strain curves of the three membranes; (**e**) TG curves of the three membranes; (**f**) remaining weights of different samples vs. the time of Fenton oxidation.

The excessive swelling of the PFSA membrane was largely confined through the incorporation of FGC. The water uptakes and swelling ratios of PFSA/G17 were significantly lower compared to those of P-PFSA and N117 (Figure 3a–c). Notably, the area swelling ratio of PFSA/G17 was just 3% at 100 °C, which is 1/20 that of P-PFSA, 1/12 that of N117 and comparable to that of the Gore-Select membrane at the same temperature. This significantly restricted swelling of PFSA/G17 is attributed to the high strength and rigidity of FGC. For the same reason, PFSA/G17 exhibited a greatly enhanced tensile strength of 81 MPa, compared with 13.7 MPa for P-PFSA and 33MPa for N117 (Figure 3d and Figure S2).

Furthermore, PFSA/G17 showed improved thermal and chemical stability relative to the commercial N117. Thermogravimetric analysis revealed that the onset temperature for sulfonate group degradation in PFSA/G17 was delayed by approximately 60 °C compared to N117 and P-PFSA membranes (Figure 3e). Additionally, the weight fraction of the FGC in the PFSA/G17 composite membrane was determined to be approximately 24 wt.% (Figure 3e). From the respective remaining weight over time for G17, PFSA/G17, N117 and PFSA in PFSA/G17 (Figure 3f), it is clear that Fenton oxidation caused severer mass loss in the case of N117 (green) than in the case of PFSA in PFSA/G17 (red), with the latter being calculated based on the mass loss of G17 and PFSA/G17. The role of FGC in mitigating PFSA degradations warrants future research.

The hydrogen gas tightness of PFSA/G17, P-PFSA and commercial Nafion 212, with all having comparable thicknesses, was evaluated by measuring the limiting current densities that are dictated by the gas permeation rate through the membrane. As summarized in Table 1, it is evident that PFSA/G17 outperformed P-PFSA and Nafion 212 in resisting H_2_ gas crossover, despite being the thinnest among the three. The improved gas tightness of PFSA/G17 can be attributed to the incorporation of FGC. The embedded FGC, as a non-permeable material, would itself block gas permeation. It would also enable the increased gas crossover resistance of the PFSA material by limiting its swelling.

## 4. Conclusions

FGC-reinforced PFSA membranes were fabricated via drop-coating the FGC with PFSA solution, followed by solvent evaporation. The reinforced membranes exhibited a substantial suppression of swelling while maintaining a low ASR. Specifically, their area swelling ratio was only ~3% at 100 °C, comparable to that of the Gore-Select membrane. Moreover, the incorporation of FGC into the electrolyte membrane led to a number of other improvements, including significantly enhanced mechanical strength, augmented resistance to gas crossover, and elevated thermal and chemical stability. This research presents a promising technological approach to developing high-performance electrolyte membranes that are key to energy storage and conversion.

## Figures and Tables

**Table 1:** A summary of the H_2_ gas crossover’s limiting current density *J_L_*, permeation flux *J_H_* and permeability *K_H_* for three membranes in a wet state at 80 °C.

Membrane	Thickness/μm	*J_L_*/mA cm^−2^	*J_H_* × 10^9^/mol s^−1^ cm^−2^	*K_H_* × 10^11^/mol cm s^−1^ cm^−2^ kPa^−1^
P-PFSA	48	0.848	4.39	10.55
PFSA/G17	25	0.281	1.46	1.82
Nafion 212	58	0.370	1.92	5.56

## Data Availability

The original contributions presented in this study are included in the article. Further inquiries can be directed to the corresponding authors.

## Supplementary Materials

The following supporting information can be downloaded at: https://www.mdpi.com/article/10.3390/membranes15060166/s1, Figure S1: (a) Plot of conductivity versus temperature for PFSA/G17, PFSA/G19 and N117; (b) Nyquist plots and fits of PFSA/G17 and N117 at 80 °C and 100% humidity; Table S1: A summary of the resistance and CPE values for PFSA/G17 and N117 membranes at 80 °C and 100% RH; Figure S2: Mechanical strength diagram of PFSA/G17, P-PFSA and N117; Figure S3: DTG diagram for PFSA/G17, N117; Figure S4: Hydrogen crossover rate of PFSA/G17, P-PFSA and N212 at 80 °C; Figure S5: Water contact angle of dry PFSA/G17, P-PFSA and N117 membranes; Figure S6: Water contact angle of wet PFSA/G17, P-PFSA and N117 membranes.
