# Supplementary material for: A Fiberglass-Cloth-Reinforced Perfluorosulfonic Acid Membrane
Source: Membranes (Basel). 2025 Jun 2;15(6):166. doi: 10.3390/membranes15060166 (PMC12194947; doi:10.3390/membranes15060166)
Supplement: Supplementary file 1 [file membranes-15-00166-s001.zip › membranes-3619304-supplementary.pdf]

Supporting Information

A Fiberglass-Cloth-Reinforced Perfluorosulfonic Acid Membrane

Zhutao Zhang [1], Yiru Dou [1], Wen Zhang [1,2,*], Li Xu [1,2] and Yuxin Wang [1,*]

[1] State Key Laboratory of Chemical Engineering and Low-Carbon Technology, Tianjin Key Laboratory of Membrane Science and Desalination Technology, School of Chemical Engineering and Technology,
Tianjin University, Tianjin 300072, China

[2] National Industry-Education Integration Platform of Energy Storage, Tianjin University, Tianjin 300072, China

* Correspondence: zhang_wen@tju.edu.cn (W.Z.); yxwang@tju.edu.cn (Y.W.)

Results and discussion

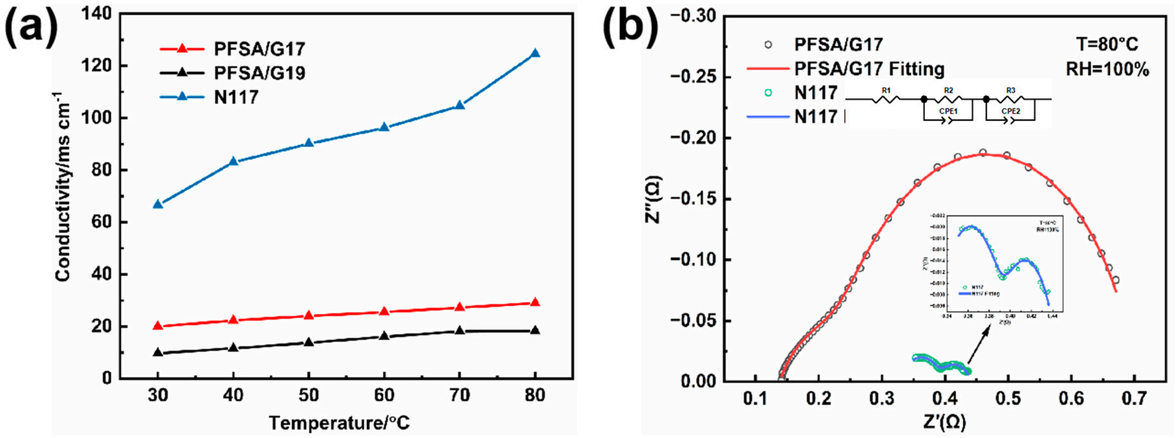

Figure S1. (a) Plot of conductivity versus temperature for PFSA/G17, PFSA/G19 and N117; (b) Nyquist plots and fits of PFSA/G17 and N117 at 80°C and 100% humidity.

Figure S1a shows the dot line plots of the conductivity of PFSA/G17, PFSA/G19 and N117 with temperature, where the conductivity is calculated based on the ASR in Figure 2a and the thickness of the membranes. The conductivity of PFSA/G17, PFSA/G19 and N117 increase with temperature, and that of PFSA/G17 is larger than that of PFSA/G19. However, the conductivities of PFSA/G17 and PFSA/G19 are smaller than that of N117,

because the non-ionic conducting FGC in the composite membrane blocks the ionic conducting channels of PFSA or increases its tortuosity, so the length of the H^+ transport path increases. However, due to the loose weaving and low thickness (about 12μm) of the selected low-gravimetric FGC, the composite membrane maintains about 30% of the conductivity of N117. In addition, the ASR of the reinforced PFSA composite membrane (PFSA/G17) with low-weight FGC can still be significantly lower than that of the commonly used commercial N117 membrane.

Figure S1b shows the Nyquist plots of PFSA/G17 and N117 at 100% relative humidity, at 80 °C. The ohmic resistance of the membrane was obtained by fitting it with Z-view software. From Figure S1b, it can be seen that the original and fitted points of PFSA/G17 and N117 overlap better. The analog circuit consists of a resistor in series with two resistors, each paralleled by a CPE. In addition, R1 in the high-frequency region represents the resistance of the membrane, and R2 and R3 in the low-frequency region represent the charge transfer resistance between the two electrodes (Pt/C). CPE1 and CPE2 represent the capacitance between the two electrodes, where CPE-T represents the capacitance and CPE-P is the similarity to the pure capacitance, usually n, which takes values of 0 to 1, with 0 being pure resistance and 1 being pure capacitance. Table S1 shows that the ohmic resistance of the PFSA/G17 membrane is 0.138 Ω, which is less than that of the N117 membrane (0.329 Ω) at 80 °C and 100% RH. The measured resistance was multiplied by the effective area (0.5 cm^2) of the membrane to calculate the area-specific resistance (ASR) at 80 °C and 100% RH, yielding values of 0.069 $\Omega \cdot cm^2$ and 0.165 $\Omega \cdot cm^2$, respectively.

Table S1. A summary of the resistance and CPE values for PFSA/G17 and N117 membranes at 80 °C and 100% RH.

Membranes	PFSA/G17	N117
R1/Ω	0.138	0.329
CPE1-T/S·cm^{-2}·s^{n}	0.0603	0.00955
CPE1-P(n)	0.641	0.699
R2/Ω	0.111	0.0622
CPE2-T/S·cm^{-2}·s^{n}	0.0831	1.15
CPE2-P(n)	0.855	0.608
R3/Ω	0.453	0.0510

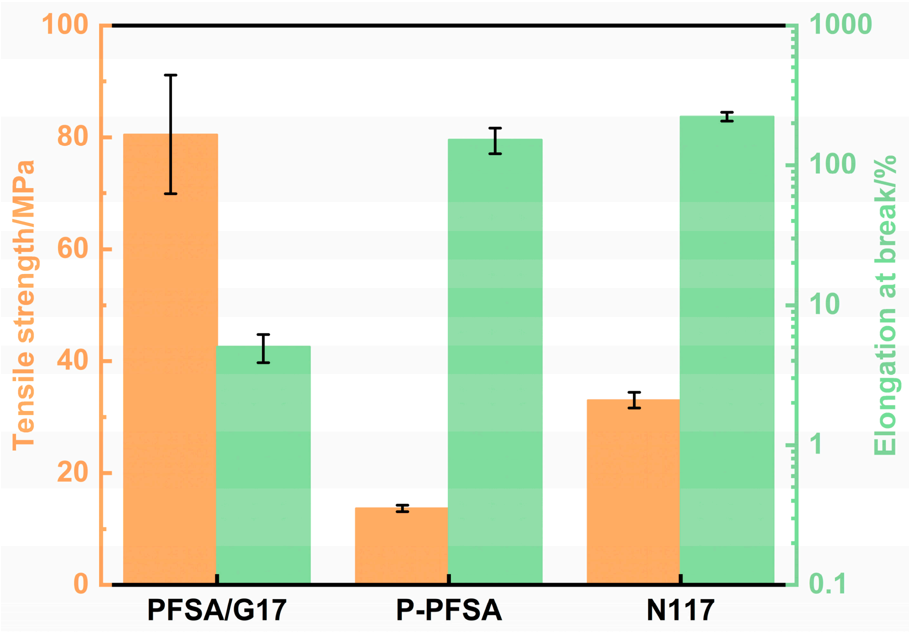

Figure S2. Mechanical strength diagram of PFSA/G17, P-PFSA and N117.

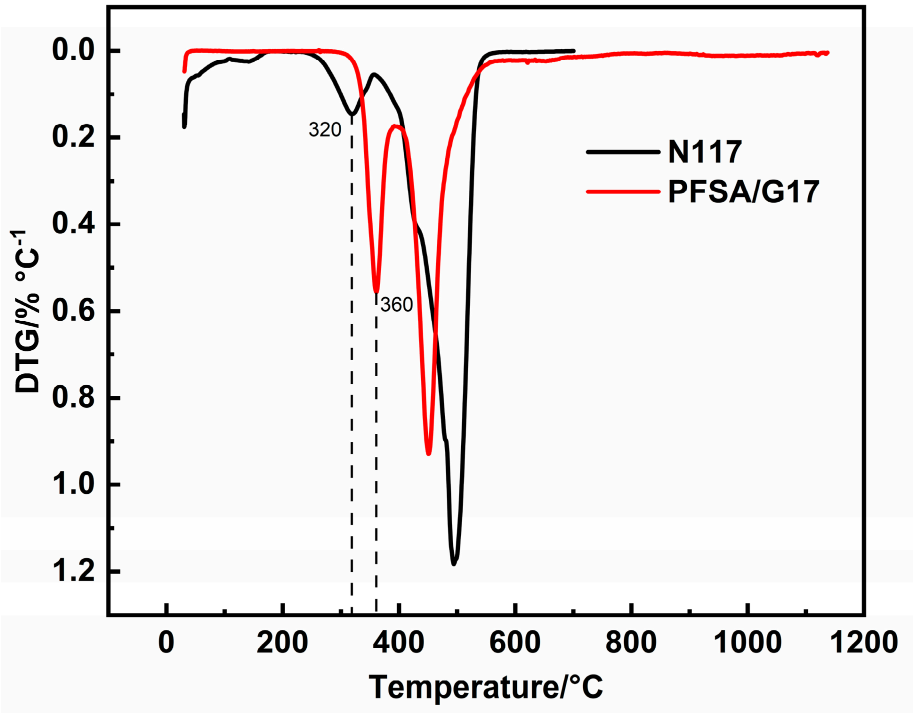

Figure S3. DTG diagram for PFSA/G17, N117.

Figure S3. shows the DTG plots of PFSA/G17 and N117 with temperature, and it can be seen that the fastest degradation of the sulfonic acid group of PFSA/G17 occurs at 360 °C, which is higher than the point at which degradation occurs for N117, which is 320 °C. Thus, the figure also shows that the composite membrane has better thermal stability than N117.

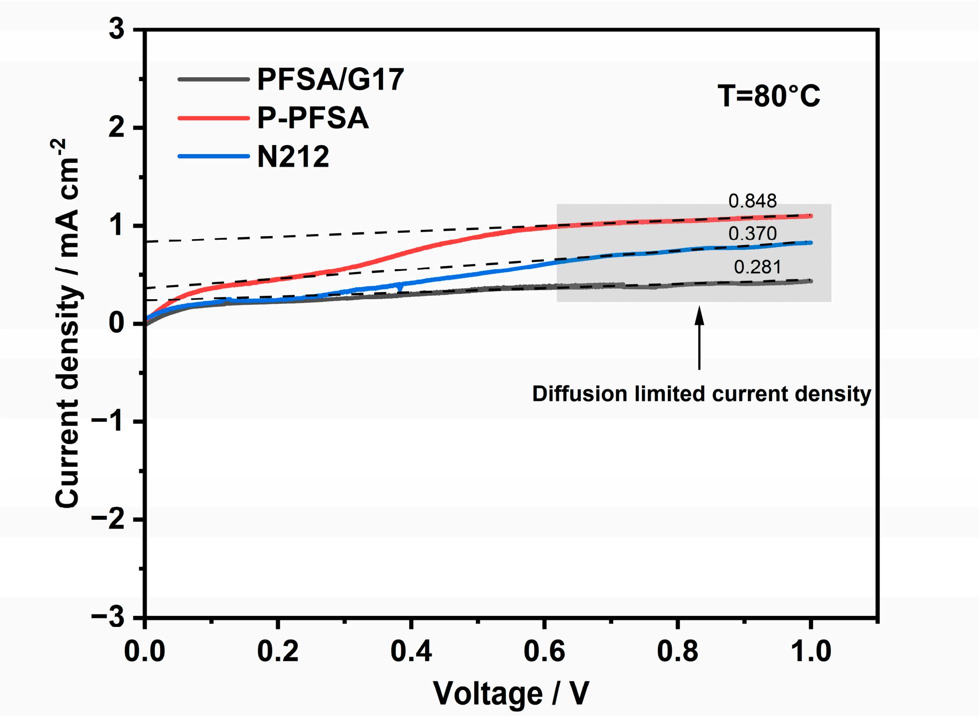

Figure S4. Hydrogen crossover rate of PFSA/G17, P-PFSA and N212 at 80 °C.

Figure S4. shows the current density–voltage curves of PFSA/G17, P-PFSA and N212 at 80 °C. From the figure, it can be seen that the current densities of the above three membranes tend to stabilize after 0.6V, indicating that they are controlled by H_2 diffusion and reach the limiting current density. A linear-fitting curve is created between 0.6 and 1.0V, and the intercept of the vertical axis corresponds to the actual HOR limit current density.

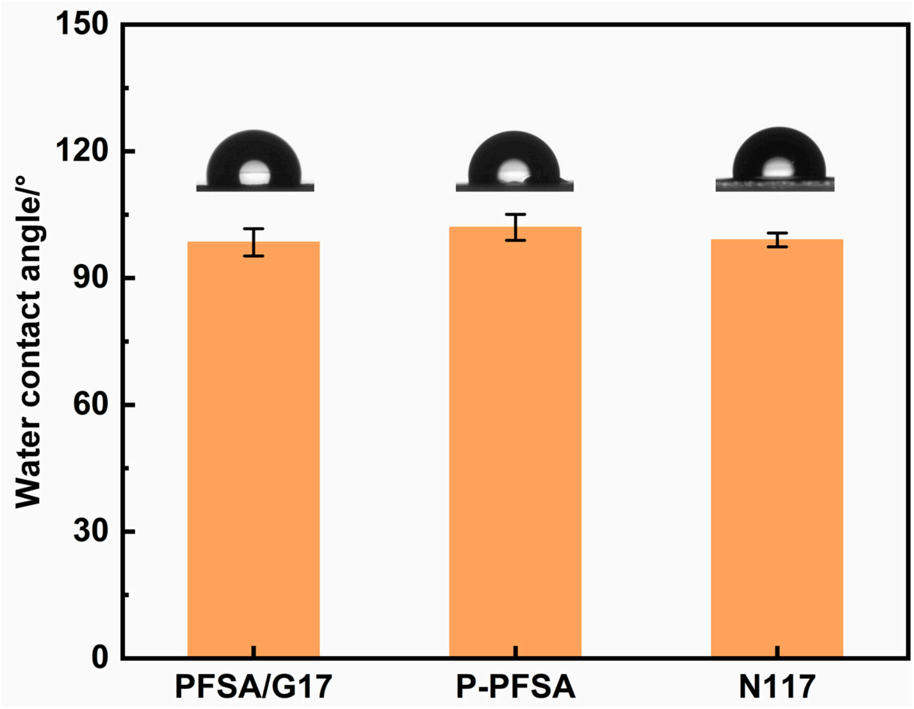

Figure S5. Water contact angle of dry PFSA/G17, P-PFSA and N117 membranes.

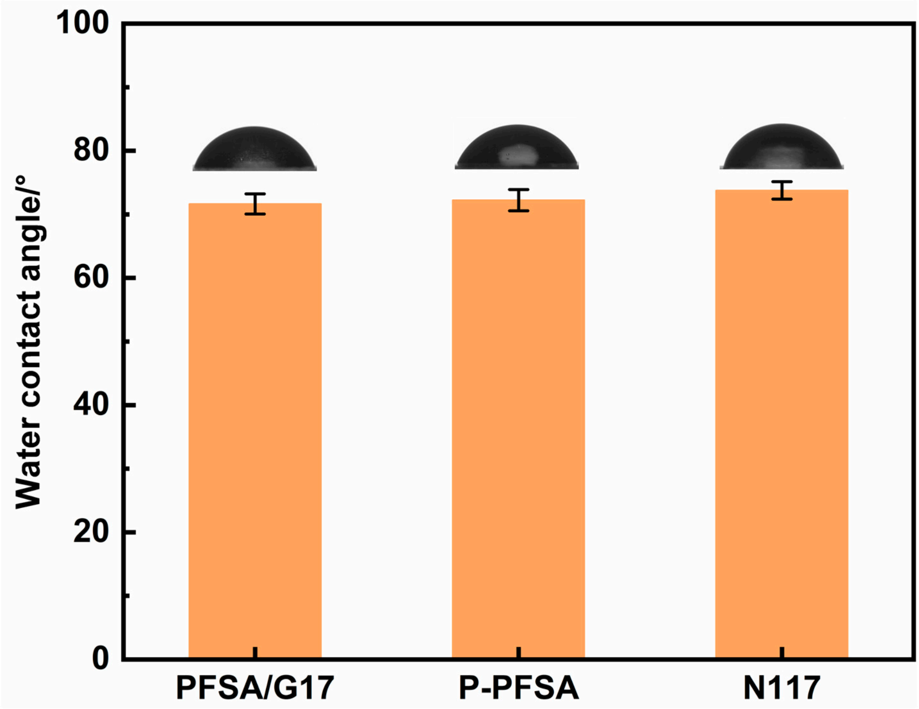

Figure S6. Water contact angle of wet PFSA/G17, P-PFSA and N117 membranes.

As shown in Figure S5, the three dry membranes exhibit similar water contact angles, all approximately 100°, indicating comparable surface hydrophobicity. This behavior can be attributed to the intrinsic hydrophobic nature of the fluorocarbon backbone in PFSA membranes. In contrast, Figure S6 reveals that the wet membranes display similar water contact angles of around 70°, demonstrating increased hydrophilicity due to enlarged hydrophilic domains. Notably, the incorporation of FGC has only a minimal influence on surface hydrophilicity.
